# Interobserver Reliability and the Sigmoid Takeoff—An Interobserver Study

**DOI:** 10.3390/cancers14112802

**Published:** 2022-06-04

**Authors:** Malene Roland Vils Pedersen, Peter Obel Otto, Chris Vagn-Hansen, Torben Sørensen, Søren Rafael Rafaelsen

**Affiliations:** 1 Department of Radiology, Clinical Cancer Centre, Vejle Hospital, University Hospital of Southern Denmark, 7100 Vejle, Denmark; peter.obel.otto@rsyd.dk (P.O.O.); chris.vagn-hansen@rsyd.dk (C.V.-H.); torson@rm.dk (T.S.); soeren.rafael.rafaelsen@rsyd.dk (S.R.R.); 2 Department of Regional Health Research, University of Southern Denmark, J.B. Winsløws Vej 19, 5230 Odense, Denmark

**Keywords:** rectal cancer, interobserver, anorectal junction, anal verge, MRI, cancer

## Abstract

**Simple Summary:**

Colorectal cancer is the second most common cancer. The sigmoid takeoff is the landmark where the colon sigmoid curves toward the sacrum viewed from sagittal magnetic resonance imaging. We assessed the interobserver variability in the assessment of the anal verge and anorectal junction in patients diagnosed with rectal cancer using magnetic resonance imaging. Our data indicate that radiologists are excellent at pointing out if a colorectal tumour is above or beneath the takeoff landmark.

**Abstract:**

**Background:** Colorectal cancer is the second most common cancer worldwide. The sigmoid takeoff is the landmark where the colon sigmoid curves toward the sacrum viewed from sagittal magnetic resonance imaging (MRI). The purpose of this study was to assess interobserver variability in the assessment of the anal verge and anorectal junction in patients diagnosed with rectal cancer on magnetic resonance imaging (MRI). **Materials and Methods:** The rectal MRI examinations were performed using a 1.5- or 3.0-tesla unit using an anterior coil and a standard scan protocol. Two senior radiologists assessed MRI scans from patients under investigation for rectal cancer. The two observers assessed the anal verge and takeoff in cm independently. Difference in agreement between the observers were evaluated using intraclass correlation (ICC) and graphically by Bland–Altman plots. **Results:** The study population (*n* = 122) included 68 (55.7%) female and 54 (44.3%) male subjects. The overall median age was 69.5 years (range 39–95 years). There was perfect agreement between the two observers when defining rectal tumor above or below the takeoff landmark. The reliability of measuring the distance from the anal verge to the sigmoid takeoff was 0.712. **Conclusion:** Overall, the study found a moderate reliability in assessing the location of the sigmoid takeoff, with a low difference in the distance measuring, as well as a good consensus concerning the determination of tumors in relation to the sigmoid takeoff. Routine implementation of this information within the report seems reasonable.

## 1. Introduction

Colorectal cancer is the second most common cancer in Europe and incidences are higher in men compared to women. In Europe, an estimation of 520,000 new cases of colorectal cancer are diagnosed annually [1]. The treatment of colorectal cancer consists of different approaches and depends on a precise and correct description of the tumor placement. To define the tumor location as either rectal or sigmoid is important and has clinical impact because treatment between the two varies.

Patients with rectal tumors undergo more frequently neoadjuvant chemo and/or radiotherapy depending on their individual risk profile as assessed on preoperative imaging, whereas patients with sigmoid cancer often have surgery alone. A definition such as the rectosigmoid junction, defined as the limit between the sigmoid colon and the rectum, is used by some. However, there has been some controversy on this subject, and there is more than one definition depending on the perspective, e.g., radiological, endoscopically, or anatomic. When using various definitions there is a potential risk of losing comparability between studies and this may be an obstacle in standardizing optimal treatment in patients with rectal cancer. Recently an international consensus Delphi study was published defining the sigmoid takeoff as the point where the colon sigmoid curves toward the sacrum viewed from sagittal magnetic resonance imaging (MRI) [2]. It is important to have a consistent accepted definition of the sigmoid takeoff.

Interobserver variability gives an important perspective on the reliability of the sigmoid takeoff determination. The skill of radiologists depends on several variables, e.g., experience, perception, and interpretation. Using different observers and independent readings will help to visualize feasible variations in interpretation. Furthermore, it is important for oncologist to know if the observer variation is caused by a progression of the disease or a measurement variation by observers [3]. Agreement is not adequately reported in radiology research as a study has pointed out [4]. Often radiology research is dedicated to diagnostic accuracy, and less attention has been given to reliability studies. Reliability studies have a high legitimacy when gold standard validation is unavailable. Guidelines for reporting reliability and agreement studies (GRRAS) has been proposed by Kottner et al. to improve the reporting in agreement studies [5].

In this study, we used the rectosigmoid junction definition based on magnetic resonance imaging (MRI) anatomy also known as the “sigmoid takeoff”. This definition has also been used in previous studies, and was first proposed by D’Souza [6]. The sigmoid takeoff is a stable landmark less affected by the tumor growth [7]. A Delphi study including international experts established that the sigmoid takeoff should be the standard definition in clinical practice [2]. Another study found the sigmoid takeoff to be a useful landmark, and found good consistency in measurement between different observers [8]. However, the literature is scarce on the interobserver reliability in MRI measurement of the distance from the anal verge to the sigmoid takeoff. It is important to emphasize that the definition of an anatomical landmark must be easily reproducible and identifiable to allow an effective surgical intervention and a reliable evaluation in numerous patients.

This study’s aim was to assess the interobserver reliability in the measurement of the anal verge and sigmoid takeoff in patients with rectal cancer and to assess the interobserver agreement on the tumor location in relation to the sigmoid takeoff.

## 2. Materials and Methods

### 2.1. Study Design

This was an interobserver study performed at the Department of Radiology, Vejle Hospital, University Hospital of Southern Denmark. The hospital has the status of Center of Clinical Excellence in treatment of colorectal cancer.

### 2.2. Patients

We conducted a retrospective study investigating a total of 122 patients. Patients were eligible for inclusion if they had an MRI investigation of the rectum performed in the period from 1 January to 31 December 2018. The inclusion criteria were an age ≥ 18 years, a diagnosis of recto-sigmoid cancer (adenocarcinoma), and no treatment or surgery initiated. A total of 33 patients with rectal polyps, 16 with follow-up investigation, 4 with colonic cancer, 2 with rectal cysts and 2 with anal cancer, and 5 with other type of cancers were excluded.

### 2.3. Observers

Two senior radiologists with more than 20 years of experience in the diagnostic of colorectal cancer participated in this study. The two observers were informed about how to perform the measurement and report the results by S.R.R by reviewing two case studies. Retrospectively, the two observers assessed the images independently, using PACS (picture archive communication system) diagnostic screens (21.3” monitor CCL358i2 from Totoku, JVCENWOOD Corporation, Kanagawa, Japan) with one month apart from each other. Furthermore, the two observers indicated if the tumor was placed above or below the takeoff landmark, respectively.

**Figure 1:**
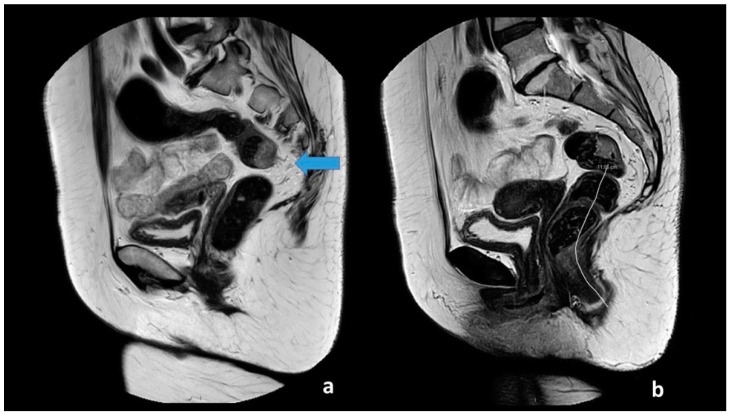
Sagittal T2-weighted images from a 65-year-old female patient with a T1 tumor located at the posterior wall just above the sigmoid takeoff. The arrow indicates the sigmoid take-off from which the sigmoid sweeps horizontally away from the sacrum and with the sigmoid blood vessels insertion (**a**). The takeoff and the lower border of the tumor is located 11 cm above the anal verge (**b**).

All images had a quality check by one of two authors (M.R.V.P and S.R.R). The two observers received a basic instruction on how to perform the assessment of the anal verge and takeoff, defined as the point from which the sigmoid sweeps horizontally away from the sacrum. The observers used the sigmoid blood vessels insertion as an adjunct in the determination of the location. The two observes measured the distance from the anal verge to the sigmoid takeoff using a curved digital ruler. The measurement of the anal verge and takeoff is visualized in Figure 1. The observers were blinded to the patient history, diagnosis, and prior image investigations. T2W-weighted MRI images with 3 mm slice thickness were used to perform the measurements.

### 2.4. MRI Imaging

The rectal MRI examinations were performed using a 1.5- or 3.0-tesla unit (Phillips Healthcare, Ingenia, Best, The Netherlands) using an anterior coil and a standard scan protocol. All patients were placed supine. A localizer scan was performed, and T2-weighted sequences in three anatomical plans, coronal, sagittal, and axial, were obtained. The images were obtained using 3 mm slices. No contrast enhancement and no oral or rectal contrast was administered, nor did the patients receive spasmolytics. The observers measured the anal verge and anorectal junction using MRI T2-weighted images in the sagittal scan plan. The MRI protocol can be seen in more detail in [9,10].

### 2.5. Ethics

The institutional review board did not require patient informed consent due to the retrospective design.

### 2.6. Statistical Analysis

Statistical calculations were performed using SPSS version 27 (IBM Corp, Armonk NY, USA). Intraclass correlation coefficient (ICC) and 95% confident intervals (CI) were calculated to compare exact measurements in cm. ICC values less than 0.50 indicate poor reproducibility, values between 0.50–0.75 indicate moderate reproducibility, values between 0.75–0.9 indicate good representability, and values greater than 0.9 indicate excellent reliability [11]. Kappa statistics were calculated for the tumor placement above or below the takeoff using the following cutoffs: <0 = poor, 0.00–0.20 = slight, 0.21–0.40 = fair, 0.41–0.61 = moderate, and 0.81–1 = almost perfect [12].

A Bland–Altman plot was used to visualize the interobserver variance from numerical values. The limit of agreement (LOA) was taken as the bias ± (1.96 S.D.) of the differences in measurement between the two observers. *p* < 0.05 was considered as the level of statistical significance.

## 3. Results

**Scheme 1:**
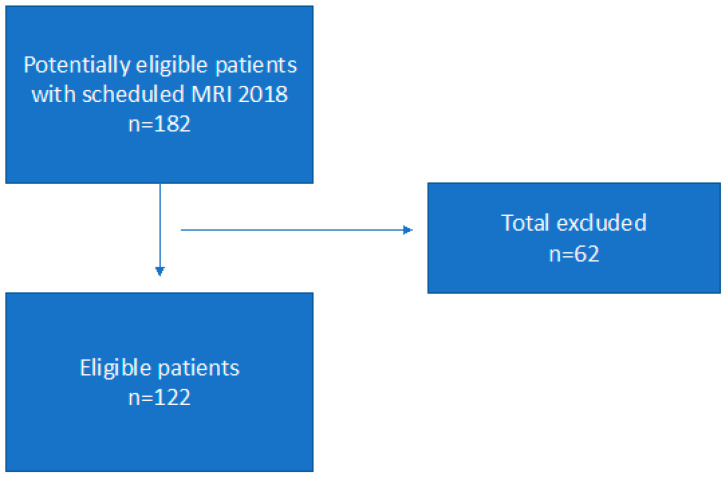
Patient flow chart.

Scheme 1 shows the patient flow. A total of 122 patients were included in the study. The study population included 68 (55.7%) females and 54 (44.3%) males all diagnosed with rectal cancer. The overall median age was 69.5 years (range 39–95 years). The median age for males was 69 (range 39–87) and for females 70 (41–95).

Observers 1 and 2 measured a mean distance from the anal verge to the takeoff of 13.3 cm CI 13.0–13.5 (SD 1.3) and 13.6 cm CI 13.3–13.8 (S.D. 1.5), respectively.

The tumor placement below or above the takeoff had perfect agreement with a perfect kappa agreement (κ = 1).

The reliability of measuring the distance from the anal verge to the takeoff had moderate reproducibility with ICC = 0.712 (CI 0.58–0.80).

**Figure 2:**
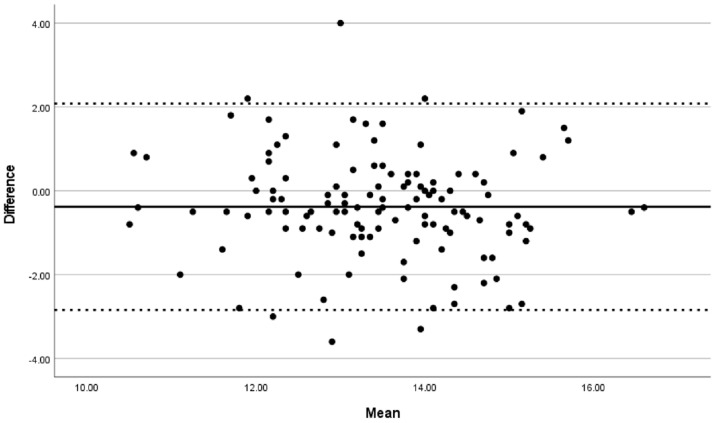
Bland–Altman plot showing the interobserver agreement. The bold line corresponds to the mean difference (−0.38 cm) and the dashed lines correspond to the upper and lower limits of agreement (95% LOA 2.08 to −2.84 cm).

The agreement between the two observers is visualized by a Bland–Altman plot in Figure 2. The Bland–Altman plot distance between the sigmoid takeoff and the anal verge showed a mean difference of −0.38 (95% LOA 2.08 to −2.84) cm (Figure 2).

## 4. Discussion

In our study the two observers were radiologists with expertise in colorectal imaging. Both observers had more than 20 years of experience in diagnostic cancer imaging. Our finding showed perfect agreement (κ = 1) when defining tumor placement above or below the sigmoid takeoff. This finding shows that the sigmoid takeoff is a reliable landmark, that can easily be implemented with high clinical value. Hazen et al. had 86 experts classify 20 rectal tumors below or above the sigmoid takeoff and found a high positive predictive value, and that training improved the agreement from 53% to 70%. That study used a various group of experts including radiologist, interns, PhD students, surgeons, and physician assistants, and not everyone was used to assess the sigmoid takeoff as a standard imaging landmark, which may have caused the low agreement (70%) [13]. Other studies have also found a high agreement in validating the sigmoid takeoff between experienced radiologists [2,14], and a study comparing CT scan with MRI images found no significant difference in the sigmoid takeoff [6].

We found a moderate reproducibility in determining the sigmoid takeoff, with a low mean difference. The Bland–Altman plots showed that it was difficult to measure the exact distance to the sigmoid takeoff in cm as the rectum is a spatial organ with intestinal bulges, fluctuations, curvatures, tortuosities, and anatomy variation. There is an individual observer measurements variation up to 5 cm (e.g., sigmoid takeoff had an LOA from 2.08 to −2.84 cm). This might explain why the LOAs were relative wide. This did not have clinical importance since the assessment of the tumor location beneath or above the point was perfect.

All tumors had a mid or high location in the rectum. The most valuable information for the surgeon is to know if the tumor is a above or below the sigmoid takeoff to prepare which operation is the most appropriate for the patient, and not necessarily the exact measurement in cm. Patients with tumors above the sigmoid takeoff are treated with surgery alone, whereas patients with rectal cancer are frequently offered neoadjuvant treatment. Moreover, D´Souza et al. highlighted that when validating the sigmoid takeoff between MRI and specimens, a tortuous rectum can be the cause for discrepancies [15]. Still, it is essential that the multidisciplinary team of surgeons, radiologist, and pathologists use a harmonized definitions of the tumor level [16].

This study has some limitations as there was no time limit for the observers to perform the measurements, and it is unknown if one observer used a lot of time on the measurements compared to the other observer. Secondly, we did not have a gold standard in this study, and it is not possible to interpret the results using a gold standard. We involved two senior observers, but if we had included more observers, e.g., some younger radiologist with less experience, we could have investigated if the more unexperienced observers also found the sigmoid takeoff to be a landmark easily implemented. However, this was not possible due to the current daily workload in our department. A recent study found the diagnostic confidence of younger radiologists increased significantly by adding CT to the MRI in the evaluation of the sigmoid takeoff [17]. Furthermore, we consider it a strength that all included patients had histopathological confirmed adenocarcinoma. It is a weakness that this study was carried out in a single department, and it is possible that a selection bias may be present. However, due to the high number of included patients this seem less likely.

## 5. Conclusions

This study demonstrated that radiologists defining tumor location above or belove the sigmoid takeoff was a reliable landmark, that can easily be implemented with a high clinical value.

## Data Availability

The data presented in this study are available on request from the corresponding author.
